# 

**Published:** 1895-11-16

**Authors:** 


**Notices of Births, Deaths, and Marriages** are inserted in this column at a charge of 2s. 6d. for an announcement not exceeding four lines.

**Notice to Advertisers and Subscribers.**

All Advertisements, Orders for Copies of The Hospital and Business Communications should be addressed to **THE MANAGER, (Not to The Editor),** The Hospital, 428, Strand, London, W.C.

The Cost of Subscription, post free, is as follows:—Per week, 2½d.; per quarter, 2s. 8d.; per half-year, 5s. 3d.; per annum, 10s. 6d. Foreign:—Per Annum, 15s. 2d.; payable, in all cases, in advance.

**NOTICE TO CORRESPONDENTS.**

All MS., Letters, Books for Review, and other matters intended for the Editor should be addressed The Editor, The Lodge, Porchester Square, London, W.

The Editor cannot undertake to return rejected MS., even when accompanied by stamped directed envelope.

**"Burdett's Hospital Annual," 1895.**

Sixth year of publication. 950 pp. Scarlet cloth, gilt lettered. Price 5s. A most complete guide to British, Colonial, Indian, and American Hospitals, Dispensaries, Nursing and Convalescent Institutions, and Asylums. A few complete sets of the preceding five issues of the "Annual" may still be obtained on application to the Publishers, The Scientific Press (Limited), 428, Strand, London, W.C.

**NOTICE.—Vol. XVIII. of "The Hospital" (April to September, 1895), in handsome cloth binding, is now on sale at the Office of this Journal. Price 6s. Cases for binding the Numbers contained in this Volume, 1s. 6d. Index to Vol. XVIII., 2½d., post free.**

**The Monthly Part for November is now ready, price 6d.; by post, 8d.**

### Scraps and Gleanings.

A sum of £6,000 was collected in Liverpool on Hospital Sunday.

A very perfectly-equipped ambulance station and vaccine laboratory is going to be erected in New York City.

The treasurer of Guy's Hospital has received a donation of £1,000 for the use of the hospital from Mr. Charles Morrison.

H.R.H. the Prince of Wales has signified his willingness to lay the foundation-stone of the new building in connection with the Sussex County Hospital.

A meeting of clergymen, representative of the various denominations in Belfast, has just been held with a view to the inauguration in the city of a "Hospital Sunday."

The contributions received during the past week towards the extension of the Edinburgh Royal Infirmary amounted to £100, including a legacy from Mr. Anderson, of Clencairn Crescent, Edinburgh.

The Co-operative Society of Accrington has given £500 towards the erection of the local cottage hospital, and Mr. George Bullough has contributed an equally generous sum to the same scheme.

At the date of the last French census there were no less than 113 genuine centenarians registered, the greater portion being men; 33 of the women were old maids, and 11 of the men bachelors.

Hospital Sunday at Birmingham was accompanied by cold weather, but the prospect of some little revival in trade was in favour of good results for the collection, the total of which realised £4,125.

The working men of Birmingham have provided an endowment of £350,000 for the hospitals of the city. In this good work they stand alone in the world; the working population of no other city has done so.

The fourth annual reunion of patients who have stayed at the Hospital Saturday Convalescent Homes at Tyn-y-Coed and Marle Hall, near Birmingham, has been held; 3,000 invitations were issued for this event.

The Saturday Fund in favour of the Lincoln County Hospital, which has lately been held, has brought in about £700 to the funds of the institution, which, as we have previously stated, is very seriously in need of financial support.

Last week the anniversary of the Derbyshire County Hospital was celebrated. Following last year's precedent, there were two services at All Saints' Church, in order to give all to an opportunity of taking part in the anniversary proceedings.

There are no fewer than three representatives of the medical profession among the French Ministry.

The number of medical men in Paris at the present time is 2,922, of whom there are 52 foreigners.

The mortality from diphtheria in London showed a marked increase during the first week in November. This was, with one exception, the highest number recorded in any week for nearly two years.

In a recently-issued statement of the Bristol branch of the Society for Prevention of Cruelty to Children it is stated that since 1889 it has investigated as many as 2,200 complaints of cruelty in Bristol and the neighbourhood, affecting 42,000 children.

Ambulances mounted on cycle wheels and driven by one or more cyclists appear to be receiving much approval. Some time back Birmingham was presented with a cycle ambulance; and an ambulance of this kind is in experimental use in Berlin also.

Her Majesty the Queen has sent contributions from Windsor Castle, and Princess Christian has lent treasures from Cumberland Lodge, for the loan exhibition in connection with the great bazaar which the Princess opened in the Town Hall, Reading, on Wednesday, in aid of the Society for the Prevention of Cruelty to Children.

The Queen has given instructions for the room in which she was born in Kensington Palace to be reinstated as far as possible as it was on May 24th, 1819, and has granted the editor of the *Gentlewoman* the privilege of sketching the room as it now appears. This interesting illustration was published by permission of Her Majesty in last week's issue of the paper.

The seventy-eighth annual meeting of the Royal Orthopædic and Spinal Hospital at Birmingham has just been held. The balance-sheet shows a slight excess of income over expenditure. The lady visitors' bazaar has benefited the funds of the hospital to the amount of £415; a second £30 has been sent from the same band of workers to the "Free Bed Fund" of this institution.

A generous offer has been made to the Tynemouth Victoria Jubilee Infirmary, Newcastle, by Mr. R. R. Donkin, M.P., who has expressed his willingness to complete the building by the erection of a new wing on the east side of the present structure and a porter's lodge, on condition that the committee acquire the land in front of the infirmary, which could be used as a garden and recreation ground for the patients.

### From A. and C. BLACK'S LIST.

**Text-Book of Operative Surgery.** By Dr. TH. KOCHER, Professor of Surgery and Director of the Surgical Clinic in the University of Bern. Translated by special permission of the Author from the Second Enlarged and Improved German Edition, by HAROLD J. STILES, M.B., C.M., Senior Demonstrator of Surgery in the University of Edinburgh; Assistant Surgeon, Royal Edinburgh Asylum for Sick Children. Illustrated with 185 cuts in text. Demy 8vo., cloth, price 20s.

**The Senile Heart.** Its Symptoms, Sequelæ, and Treatment. By GEO. W. BALFOUR, M.D., LL.D., Consulting Physician to the Royal Infirmary and to the Royal Public Dispensary, Edinburgh. Crown 8vo., cloth, price 5s.

**Plea for a Simpler Life.** By GEORGE S. KEITH, M.D., F.R.C.P.E. Crown 8vo., cloth, price 2s. 6d.

**Text-Book of General Pathology and Pathological Anatomy.** By Professor RICHARD THOMA, of Dorpat. Translated by ALEX. BRUCE, M.D., Lecturer on Pathology, Surgeons' Hall, Edinburgh. [*Vol. I. in preparation.*

A. AND C. BLACK, Soho Square, London, W.

Just out.
NEW REPORTS OF THE JOHNS HOPKINS UNIVERSITY, BALTIMORE.

**The Malarial Fevers of Baltimore.**—An Analysis of 616 Cases of Malarial Fever, with Special Reference to the Relations existing between different Types of Hæmatozoa and different Types of Fever. By WM. SYDNEY THAYER, M.D., and JOHN HEWETSON, M.D., Assistants in the Medical Clinic of The Johns Hopkins Hospital. Price 10s net.

**A Study of some Fatal Cases of Malaria.**—By LEWELLYS F. BARKER, M.B.Tor., Associate in Anatomy Johns Hopkins University, and Assistant Resident Pathologist the Johns Hopkins Hospital. Price 3s. 9d. net.

Being the first parts of Vol. V. (now in progress) of The Johns Hopkins Hospital Reports.

Sole Agents for the United Kingdom,
THE SCIENTIFIC PRESS (LIMITED), 428, Strand, London, W.C.

### "THE HOSPITAL"

AN INDEPENDENT JOURNAL OF

**The Medical Sciences**

AND

**Hospital Administration,**

WITH WHICH IS ISSUED WEEKLY

A SPECIAL

### SUPPLEMENT FOR NURSES.

PUBLISHED EVERY SATURDAY.

PRICE TWOPENCE.

Matrons, Sisters, or Nurses will find in THE HOSPITAL a chronicle of the latest Nursing News, Practical Articles of the highest value to those who wish to keep themselves abreast with the times, and brightly written articles from Nurses all over the world.

SUBSCRIPTIONS CAN COMMENCE AT ANY TIME.

### TERMS OF SUBSCRIPTION.

### WEEKLY ISSUE.

| | Great Britain. | Foreign and Colonial. |
|---|---|---|
| For One Year ... ... | 10s. 6d. ... | 15s. 2d. |
| For Six Months ... | 5s. 3d. ... | 7s. 7d. |
| For Three Months... | 2s. 8d. ... | 3s. 10d. |

### MONTHLY PARTS.

| | Post Free to any Part of the World. |
|---|---|
| For One Year ... ... | 8s. 6d. |
| For Six Months ... ... | 4s. 3d. |
| For Three Months ... ... | 2s. 2d. |

Postal Orders and Cheques should be made payable to "The Scientific Press, Limited."

N.B.—In order to prevent delay, all business communications should be addressed to "THE MANAGER" only, and not to any person individually.

### CHANGE OF ADDRESS.

In event of change of address, notice should be forwarded to the Publishers by the Saturday previous to date of publication, and the old address should also be given.

"THE HOSPITAL" can be obtained of all Booksellers and Newsagents in the United Kingdom, and at all the Railway Stalls, of Messrs. W. H. Smith and Son; and also from the following Agents in the Colonies and Abroad:—

PARIS: Librairie Galignani, 224, Rue de Rivoli.
BERLIN: Messrs. A. Asher and Co.
LEIPSIG: Mr. F. A. Brockhaus.
NEW YORK: The International News Co.
MONTREAL: The Montreal News Co.
TORONTO: The Toronto News Co.
MELBOURNE, SYDNEY, ADELAIDE, and NEW ZEALAND: Messrs. Gordon and Gotch.
INDIA: At all the Railway Bookstalls and Agencies of Messrs. A. H. Wheeler and Co.
SOUTH AFRICA: Messrs. Juta, Cape Town.

PUBLISHING OFFICES: 428, STRAND, LONDON, W.C.

### PAMPHLETS ON NURSING.

No. 2, Now Ready, price 1½d. per copy, or 1s. per dozen.

**A GREAT MOVEMENT—THE NURSES' CO-OPERATION.**

By Mrs. FURLEY SMITH.

Also No. 1, price 1½d. per copy, or 1s. per dozen.

**THE ADVANTAGES AND PRIVILEGES OF A TRAINED DISTRICT NURSE, AND THE DUTIES OF THE DISTRICT TOWARDS HER.**

By HENRY C. BURDETT.

London: THE SCIENTIFIC PRESS (Limited), 428, Strand, W.C.

### STANDARD TEXT-BOOKS ON NURSING, &c.

Now Ready. With numerous Illustrations, demy 8vo., blue buckram, gilt, 3s. 6d.

### DIET IN SICKNESS AND IN HEALTH.

### By Mrs. ERNEST HART,

*Formerly Student of the Faculty of Medicine of Paris, and of the London School of Medicine for Women.*

With an Introduction by Sir HENRY THOMPSON, F.R.C.S., M.B. Lond.

"Does not ask the reader blandly to accept such and such a diet for such and such complaint, but explains to him, briefly and simply, the why and wherefore, and gives in each case excellent physiological lessons, illustrated with comprehensive diagrams. Another good point is its cheerful sensible tone. The book is excellent in every way, and may be warmly recommended to the enormous number of people who, for one reason or another, have to be careful about their diet."—*Westminster Gazette.*

"Mrs. Hart not only expounds the principles of dietetics with insight and authority, applying them particularly and generally to all forms of disease and disability, but she is also able to act as a guide to the actual practice of dietetics. A really useful handbook to all invalids, nurses, medical students, and their kind."—*Science Siftings.*

Third and Revised Edition. Demy 8vo., 209 pp., profusely Illustrated with Copyright Portraits and Drawings. Price 2s. 6d.

**HOW TO BECOME A NURSE**: And How to Succeed. A complete guide to the Nursing Profession for those who wish to become Nurses, and a useful book of Reference for Nurses who have completed their training and seek employment. Compiled by HONNOR MORTEN (Author of "Sketches of Hospital Life," "The Nurse's Dictionary," &c.)

Second and Revised Edition. Demy 16mo., suitable for the apron pocket, handsomely bound in cloth, 140 pp. Price 2s.; in handsome leather, gilt, price 2s. 6d. net.

**THE NURSES' DICTIONARY OF MEDICAL TERMS AND NURSING TREATMENT.**—Compiled for the use of Nurses by HONNOR MORTEN. Containing descriptions of the principal Medical and Nursing Terms and Abbreviations, Instruments, Drugs, Diseases, Accidents, Treatments, Physiological Names, Operations, Foods, Appliances, &c., &c., encountered in the Ward or Sick-room.

Second Edition. Enlarged, demy 8vo., profusely illustrated, nearly 200 pp., cloth. Important Text-book for Asylum Attendants. Price 2s. 6d.

**MENTAL NURSING.** A text-book specially designed for the instruction of Attendants on the Insane. By WILLIAM HARDING, M.B. The want of a complete book for the instruction of asylum attendants has been long felt, and it is with confidence that the publishers recommend this work to the managers of all Institutions for the Insane.

200 pp., Crown 8vo., cloth, 3s. 6d. Profusely Illustrated with Original Drawings, with list of Instruments required, and a Glossary of Terms.

**OPHTHALMIC NURSING.** By SYDNEY STEPHENSON, M.B., F.R.C.S.E., Surgeon to the Ophthalmic School, Hanwell, W., &c., &c. This is a valuable contribution to Nursing literature on a subject hitherto never handled with special reference to Nursing. On account of its compact and clear arrangement, and its numerous illustrations, it is specially recommended to the use of the Nursing Profession.

Crown 8vo., imitation cloth, 5s. With 10 plates and many Illustrations in the Text, and Fac-simile Autograph Letters from the late Sir Andrew Clark, M. Pasteur, &c.

**INFANT FEEDING BY ARTIFICIAL MEANS**: A Scientific and Practical Treatise on the Dietetics of Infancy. By S. H. SADLER, Author of "Suggestions to Mothers," "Management of Children," "Education."

Crown 8vo., cloth gilt, price 2s. 6d.

**THE MOTHER'S HELP AND GUIDE TO THE DOMESTIC MANAGEMENT OF CHILDREN.** By P. MURRAY BRAIDWOOD, M.D., formerly Senior Medical Officer to the Wirral Hospital for Sick Children.

Crown 8vo., cloth gilt, containing many Plates and Illustrations, price 2s. 6d.

**SPINAL CURVATURE AND AWKWARD DEPORTMENT**: Their Causes and Prevention in Children. Dr. GEORGE MULLER, Professor of Medicine and Orthopædics, Berlin. English Edition, edited and adapted by RICHARD GREENE, F.R.C.P.

"An able essay upon the effect produced by exercise, attitude, and movement upon the growth and development of the body. He further gives directions which any intelligent parent or teacher could carry out with the help of very simple apparatus, showing how to avoid or, in early cases, to cure certain malformations which, in the majority of cases, arise either from the neglect of very simple hygienic rules or from carelessness."—*Daily Chronicle.*

Demy 8vo., nearly 200 pp., cloth gilt, profusely illustrated with over 70 special cuts, price 6s.

**THE ART OF MASSAGE.** By A. CREIGHTON HALE. A complete text-book of this valuable art. The most practical, simple, and generally useful work published on the subject. With fully illustrated chapters on the Anatomy of the Human Body.

Important New Handbook of Midwifery. Handsomely bound in leather 260 pp., profusely illustrated, price 6s.

**A PRACTICAL HAND-BOOK OF MIDWIFERY.** By FRANCIS W. N. HAULTAIN, M.D. A Practical Manual produced in a portable and convenient form for reference.

Third Edition, enlarged and partly re-written, crown 8vo., cloth gilt, about 200 pp., price 2s. 6d.

**HOSPITAL SISTERS AND THEIR DUTIES.** By EVA C. E. LUCKES, Matron to the London Hospital. The favourable reception accorded to the first two editions of this useful and interesting work, and the regular demand for copies of it, encourage the publishers to place this third edition, greatly improved and enlarged, before the Institutions. The experience and position of its author entitle it to authority, and every Matron, Sister, or Nurse who aspires to become a Sister should read the advice and information contained in its pages.

Demy 8vo., cloth gilt, 260 pp. Price 3s. 6d.

**ART OF FEEDING THE INVALID.** By a MEDICAL PRACTITIONER and a LADY PROFESSOR OF COOKERY. A popular treatise on the most frequent disorders; with detailed lists of suitable diet and original recipes for daily use. This important work has been undertaken to supply a want long felt by Matrons and Lady Superintendents in Hospitals and Institutions of all kinds where invalids are treated, as well as in private households. It is of the greatest value to nurses in private practice.

Demy 8vo., 200 pp., cloth boards, copiously Illustrated, price 3s. 6d.

**SURGICAL WARD-WORK AND NURSING.** By ALEXANDER MILES, M.D. (Edin.), C.M., F.R.C.S.E. A practical manual of clinical instruction for Nurses and Students in the Wards. Concisely though comprehensively treated.

Crown 8vo., 500 pp., Second Edition, 7 Plates, 18 Illustrations, Charts, &c., price 7s. 6d. net.

**NURSING.** By ISABEL ADAMS HAMPTON.

"It is not too much to say that in no single volume yet published has the subject been so scientifically, carefully, and completely treated as it has been by Miss Hampton. . . . Whether as a teaching manual or as a book of reference, the thoroughly practical instruction which it conveys is sure to obtain a wide circulation."—*The Hospital.*

Demy 8vo., cloth extra, price 3s. 6d. (post free). Popular edition, stitched, price 1s. 6d.

**OUTLINES OF INSANITY.** A Popular Treatise on the Salient Features of Insanity. By FRANCIS H. WALMSLEY, M.D., Medical Superintendent of the Darenth Asylum, Metropolitan Asylums Board, Member of Council of Medico-Psychological Association.

A clearly written manual in which the various features of mental disorder are fully expounded. The author's style is interesting and the reader's attention is riveted throughout.

Crown 8vo., cloth gilt, Illustrated. price 5s.

**HELPS IN SICKNESS AND TO HEALTH**: Where to Go and What to Do. Being a Guide to Home Nursing and a Handbook to Health in the Habitation, the Nursery, the School-room, and the Person, with a chapter on Pleasure and Health Resorts. By HENRY C. BURDETT, Author of "Hospitals and Asylums of the World," "Pay Hospitals of the World," "Cottage Hospitals, General, Fever, and Convalescent," &c., &c.

"It would be difficult to find one which should be more welcome in a household than this unpretending but most useful book."—*The Times.*

In the Press. Ready immediately. Greatly Enlarged and entirely Revised Edition. Profusely Illustrated. Crown 8vo., cloth. Price 3s. 6d.

**NURSING: ITS THEORY AND PRACTICE.** By PERCY G. LEWIS, M.D., M.R.C.S. Being a complete Text-book of Medical, Surgical, and Monthly Nursing.

This new edition is enlarged by over 50 pp., with special new chapters on monthly nursing and confinements.

LONDON: THE SCIENTIFIC PRESS (LIMITED), 428, STRAND, W.C.

